# A Novel Error Correction Approach to Improve Standard Point Positioning of Integrated BDS/GPS

**DOI:** 10.3390/s20216162

**Published:** 2020-10-29

**Authors:** Luyao Du, Jing Ji, Zhonghui Pei, Wei Chen

**Affiliations:** 1 School of automation, Wuhan University of Technology, Wuhan 430070, China; duluyao@whut.edu.cn; 2 School of information engineering, Wuhan University of Technology, Wuhan 430070, China; jijingisme@whut.edu.cn (J.J.); peizhonghui@whut.edu.cn (Z.P.)

**Keywords:** deep learning, positioning, error correction

## Abstract

To improve the standard point positioning (SPP) accuracy of integrated BDS (BeiDou Navigation Satellite System)/GPS (Global Positioning System) at the receiver end, a novel approach based on Long Short-Term Memory (LSTM) error correction recurrent neural network is proposed and implemented to reduce the error caused by multiple sources. On the basis of the weighted least square (WLS) method and Kalman filter, the proposed LSTM-based algorithms, named WLS–LSTM and Kalman–LSTM error correction methods, are used to predict the positioning error of the next epoch, and the prediction result is used to correct the next epoch error. Based on the measured data, the results of the weighted least square method, the Kalman filter method and the LSTM error correction method were compared and analyzed. The dynamic test was also conducted, and the experimental results in dynamic scenarios were analyzed. From the experimental results, the three-dimensional point positioning error of Kalman–LSTM error correction method is 1.038 m, while the error of weighted least square method, Kalman filter and WLS–LSTM error correction method are 3.498, 3.406 and 1.782 m, respectively. The positioning error is 3.7399 m and the corrected positioning error is 0.7493 m in a dynamic scene. The results show that the LSTM-based error correction method can improve the standard point positioning accuracy of integrated BDS/GPS significantly.

## 1. Introduction

Over the past few years, satellite navigation technology has achieved rapid development, but there are still some problems to be solved in some aspects. For example, in terms of reliability and integrity, a single navigation system is difficult to meet the requirements of positioning accuracy in some areas with complex terrain due to insufficient visible satellites [1]. In addition, in some applications with high dynamic requirements, the dynamic positioning of satellite navigation is not good enough to meet the needs of practical applications. Therefore, to improve navigation positioning accuracy and performance, the integration of GPS and BDS can be used, which will increase the number of visible stars; enhance the quality of observation; reduce the spatial position accuracy factor; improve the integrity, reliability and stability of the satellite navigation system; improve the ability of satellite navigation system positioning services and the positioning accuracy of the integrated positioning system; and expand the application field of satellite navigation [2].

Many methods can be used to achieve the positioning of integrated BDS/GPS, such as real-time kinematic (RTK), precision point positioning (PPP) and SPP. RTK is a technique that uses the carrier phase in the satellite signal as the observation and uses the difference equation to solve the ambiguity to achieve the dynamic positioning between the mobile station and the reference station [3]. At present, the static positioning accuracy of RTK technology can reach even centimeters or even millimeters [4]. However, due to the dependence of RTK technology on carrier signals, it is susceptible to signal spoofing, interference and occlusion. PPP is a solution to solving the position using precise ephemeris, precise satellite orbit and precision satellite clock and dual-frequency carrier phase observations [5,6]. It can be divided into non-differential PPP and differential PPP according to the data processing [7,8,9]. The current PPP positioning accuracy can reach decimeter or even centimeter level [10]. However, the shortcoming of PPP is that the initialization time and re-initialization time after the satellite loses lock is very long, and the precision ephemeris can only be processed afterwards, so its scope of use is limited. SPP is a technique for determining the absolute coordinates of the receiver in Earth’s coordinate system based on satellite broadcast ephemeris and observations from a single receiver [11,12]. Due to the above shortcomings of RTK and PPP, if the positioning accuracy of SPP can be improved, it would be more suitable for use in vehicle applications.

There are many error sources in the positioning process [13,14,15]. In the positioning process, various errors are roughly divided into the following three aspects according to different sources: (a) Satellite-related errors, which mainly include satellite clock errors and ephemeris errors, are due to the fact that they are affected by various complex factors, making it difficult to accurately match the operational model to the correction model and orbital parameters. (b) There are errors related to signal propagation. The influence of various substances in the atmosphere that satellite signals must pass through the atmosphere is called atmospheric delay. Atmospheric delays generally include ionospheric delays and tropospheric delays. (c) Receiver-related errors mainly include multi-path effects, electromagnetic interference, receiver noise, and software calculation errors. To achieve precise positioning, a correction model of the positioning error must be established to correct the positioning accuracy and minimize the effects of errors.

Different methods have been proposed by many researchers to reduce positioning errors. Ke Han et al. proposed a wavelet packet algorithm based on two-dimensional moving weighted average processing (WP-TD) for extracting multipath [16]. Hailiang Xiong et al. presented a novel hybrid GPS/INS/Doppler velocity log (DVL) positioning method and a new robust adaptive federated strong tracking Kalman filter (RAFSTKF) algorithm for data fusion [17]. An improved robust adaptive Kalman filtering algorithm was proposed by Qieqie Zhang et al., which includes a classification robust equivalent weight function model based on t-test statistic [18]. Most of them focus only on reducing the error of one or several sources, but not reducing the error of multiple sources.

Recently, deep learning has become one of the most active technologies in many research areas. Deep learning usually refers to stacking multiple layers of neural network to perform machine learning tasks [19], which can provide a different level of abstraction to improve the learning ability and task performance [20]. Recurrent neural network (RNN) with Long Short-Term Memory (LSTM), which was originally introduced by Hochreiter et al. [21], has emerged as an effective and scalable model for several learning problems related to sequential data [22,23,24,25]. Since the SPP solution process is also a sequential data problem, the LSTM network can be used to reduce the error caused by multiple sources, including satellite clock error, ephemeris error, ionosphere and tropospheric delays, multi-path effect and the receiver error.

In this paper, a novel standard point positioning approach to integrate BDS/GPS (all GNSS systems would be applicable), which uses the learning method to predict the multi-source error as a whole, is proposed to reduce the multiple sources errors. The contributions of this paper can be summarized as follows:

- The SPP calculation model of Integrated BDS/GPS is implemented based on the BDS/GPS original ephemeris file and observation file data, which are collected through Sinan M300 GNSS receiver.
- The LSTM-based error correction method is proposed and implemented combined with the traditional filtering method to reduce the multiple sources errors, in which the LSTM recurrent neural network is used to predict the positioning error of the next epoch, so as to reduce the positioning error at the receiving end.
- Experiments in static and dynamic scenarios were conducted on the data collected by Sinan M300 GNSS receiver and the result of proposed approach was compared with the traditional positioning methods, which proved that the proposed approach can improve the standard point positioning performance of integrated BDS/GPS significantly.

The rest of the paper is organized as follows. In Section 2, the method of standard point positioning of integrated BDS/GPS is described. The LSTM-based error correction method is detailed in Section 3. Section 4 presents the experimental results, including the WLS method, Kalman filter, WLS–LSTM error correction method and Kalman–LSTM error correction in a static scene and the correction results in a dynamic scene. Finally, conclusions are given in Section 5.

## 2. Standard Point Positioning of Integrated BDS/GPS

### 2.1. Unification of Time and Space Benchmarks

Both the BDS and GPS time systems use an atomic time base with consistent seconds, both of which are counted in weeks and seconds. The time reference of the BDS is Bei Dou navigation satellite system time (BDT), whose starting epoch is the coordinated universal time (UTC) of 00:00:00 on 1 January 2006. The time reference of the GPS is GPS time (GPST), whose starting epoch is the UTC of 00:00:00 on 6 January 1980. Their different time bases determine the time of each other, but both can be linked to UTC. The conversion relationship between BDT and GPST can be defined as [26]:

(1) $$\begin{array}{cc} \; & BDT\;\mathrm{week}=GPST\;\mathrm{week}+1356\\ \; & BDT\;\sec=GPST\;\sec+14 \end{array}$$

BDS adopts China geodetic coordinate system 2000 (CGCS2000), while GPS adopts world geodetic coordinate system 1984 (WGS-84). The coordinate frames of both systems are the International Terrestrial Reference Frame (ITRF) defined by the International Earth Rotation Service (IERS) organization. In terms of coordinate system accuracy, the difference between the CGCS2000 and ITRF is about 5 cm, while the accuracy of the WGS-84 has reached 2 cm, which is basically consistent with the accuracy of ITRF. The study has proved that the coordinate and gravity changes caused by the flatness difference of the two coordinate systems are negligible compared with the current high-precision measurement level. The WGS84 coordinate system and the CGCS2000 coordinate system are compatible at the level of accuracy for standard point positioning.

### 2.2. Integrated Positioning Model

For GPS satellites, the observation equation for pseudorange is:

(2) $$P^{G}=\rho^{G}+c\left(dt_{G}-dt^{G}\right)+T^{G}+I^{G}+e^{G}$$

where *P${}^{G}$* is the pseudorange measurement from satellite to receiver; $\rho^{G}$ represents the geometric distance from receiver to satellite; *c* denotes the speed of light; *dt${}_{G}$* means the receiver clock error; *dt${}^{G}$* is the star clock error; *T${}^{G}$*, *I${}^{G}$* and *e${}^{G}$* represent the tropospheric delay, ionospheric delay and other delay error terms, respectively; and *G* stands for GPS satellites.

For BDS satellites, the observation equation for pseudorange is:

(3) $$P^{C}=\rho^{C}+c\left(dt_{C}-dt^{C}\right)+T^{C}+I^{C}+e^{C}$$

where *C* stands for BDS satellites and the meaning of the parameters is the same with the GPS satellites.

Suppose the number of GPS satellite signals received by the receiver is *m* and the number of BDS satellite signals is *n*. The mathematical model of the combined positioning is shown in Equation (4) [27].

(4) $$\begin{array}{c} AX=\left[\begin{array}{ccccc} -\frac{X^{G1}-X_{i0}}{\rho_{i}^{G1}} & -\frac{Y^{G1}-Y_{i0}}{\rho_{i}^{G1}} & -\frac{Z^{G1}-Z_{i0}}{\rho_{i}^{G1}} & 1 & 0\\ \vdots & \vdots & \vdots & \vdots & \vdots \\ -\frac{X^{Gm}-X_{i0}}{\rho_{i}^{Gm}} & -\frac{Y^{Gm}-Y_{i0}}{\rho_{i}^{Gm}} & -\frac{Z^{Gm}-Z_{i0}}{\rho_{i}^{Gm}} & 1 & 0\\ -\frac{X^{C1}-X_{i0}}{\rho_{i}^{C1}} & -\frac{Y^{C1}-Y_{i0}}{\rho_{i}^{C1}} & -\frac{Z^{C1}-Z_{i0}}{\rho_{i}^{C1}} & 0 & 1\\ \vdots & \vdots & \vdots & \vdots & \vdots \\ -\frac{X^{Cn}-X_{i0}}{\rho_{i}^{Cn}} & -\frac{Y^{Cn}-Y_{i0}}{\rho_{i}^{Cn}} & -\frac{Z^{Cn}-Z_{i0}}{\rho_{i}^{Cn}} & 0 & 1 \end{array}\right]\left[\begin{array}{c} \Delta X_{i}\\ \Delta Y_{i}\\ \Delta Z_{i}\\ c\cdot dt_{Gi}\\ c\cdot dt_{Ci} \end{array}\right]=b \end{array}$$

where *X${}^{k}$* denotes the X-axis coordinate of satellite *k*, *Y${}^{k}$* denotes the Y-axis coordinate of satellite *k* and *Z${}^{k}$* denotes the Z-axis coordinate of satellite *k*. $\rho_{i}^{k}$ means the geometric distance from receiver *i* to satellite *k*. *X${}_{i0}$*, *Y${}_{i0}$* and *Z${}_{i0}$* represent initial estimated coordinates of the receiver *i* on X-axis, Y-axis and Z-axis, while $\Delta X_{i}$, $\Delta Y_{i}$ and $\Delta Z_{i}$ stand for the difference between estimated coordinates and real coordinates, respectively.

If *m + n ≥ 5* in the formula, the value of $\Delta X_{i}$, $\Delta Y_{i}$ and $\Delta Z_{i}$ can be calculated by least square method, iteratively iterating to minimize *b*, where *b = P${}_{i}$ − $\rho_{i}$ + c·dt${}^{G}$ + c·dt${}^{C}$ − T${}_{i}$ − I${}_{i}$ − e${}_{i}$*.

The steps of Integrated BDS/GPS positioning are as follows:

1. Read RINEX format file generated by Sinan M300 GNSS receiver separately. In the RINEX format file, N file represents GPS ephemeris file, C file represents BDS ephemeris file and O file represents BDS/GPS observation file.
2. Convert the UTC time of BDS and GPS in the observation file into BDS time and GPS time, and unify the time.
3. Judge the number of visible satellites in a certain epoch. If the number of visible satellites is greater than or equal to 5, continue, and, if not, the end.
4. Select the effective ephemeris. The reference time of the effective ephemeris must be within 2 h of BDS/GPS time.
5. Calculate position and clock difference of BDS/GPS respectively. Then, correct error of Earth’s rotation.
6. Calculate the elevation and azimuth of the satellite using the position of the satellite and the receiver.
7. Use the error correction model to calculate the corresponding ionosphere and tropospheric delays.
8. Calculate receiver position until the difference between the two positions of the receiver is less than a threshold.
9. Perform WLS [28,29] or Kalman filter [29,30,31] on positioning.

## 3. Error Correction Method

### 3.1. The LSTM Model

**Figure 1:**
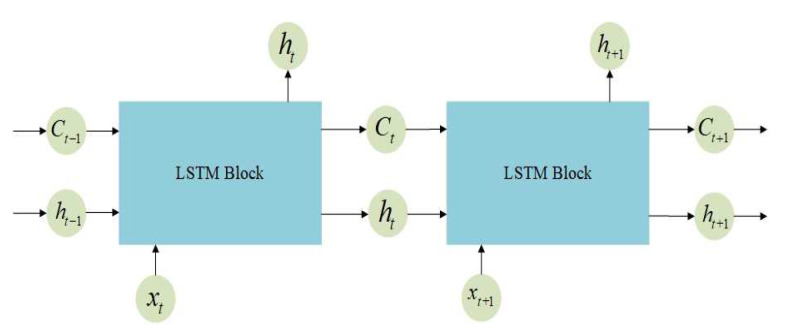
The un-rolled LSTM sequential architecture.

LSTM network is a special RNN network, which also has a module link structure, but the difference is the module structure of the hidden layer [32]. The input of the LSTM network at time *t* includes not only the input value of the current time network, but also the output at time *t−1* and the unit status at time *t−1*. At the same time, its output not only has the output of the current moment, but also the unit state of the current moment. The structure of un-rolled LSTM sequential architecture is shown in Figure 1.

**Figure 2:**
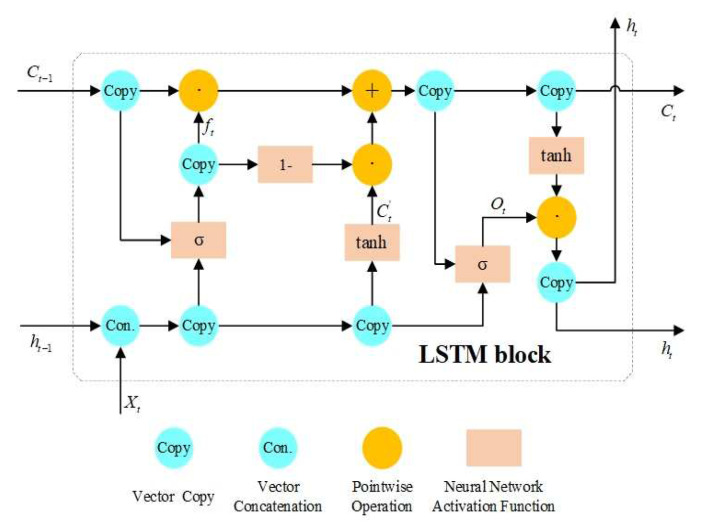
The structure of an LSTM block.

In this paper, LSTM recurrent neural network with peephole is performed on the error correction of integrated BDS/GPS point positioning. The structure of an LSTM block is shown in Figure 2.

Compared to the standard four-layer LSTM neural network module, the LSTM recurrent neural network with the peephole has only three neural network layers, including two sigmoid layers and one tanh layer. At the same time, forget gates are coupled with input gates in the modified LSTM recurrent neural network.

In the modified LSTM block, the input of the forget gate layer consists of three vectors, which are the state of the memory cell at the previous moment ($C_{t-1}$), the output of the memory cell at the previous moment ($h_{t-1}$) and the input of the memory cell at the current moment ($X_{t}$) [33]. $W_{f}$, $b_{f}$ and $f_{t}$ are used to represent the weight, offset and output vector of sigmoid neural network layer of the forget gate, respectively. The sigmoid activation function can be defined as:

(5) $$\sigma \left(x\right)=\frac{1}{1+e^{-x}}$$

The output vector of forget gate layer can be defined as:

(6) $$f_{t}=\sigma \left(W_{f}\times C_{t-1}+W_{f}\times h_{t-1}+W_{f}\times X_{t}+b_{f}\right)$$

The input gate layer controls the information injected into the memory cells by coupling with the forget gate layer to determine the update of the cell state. *C${}_{t}^{{}^{\prime }}$* is used to denote the vector of new information to be injected into the memory cell, which is the output of the tanh layer. The weights and offsets of the tanh layer are represented by *W${}_{c}$* and *b${}_{c}$*. The tanh activation function can be defined as:

(7) $$tanh\left(x\right)=\frac{1-e^{-2x}}{1+e^{-2x}}$$

The *C${}_{t}^{{}^{\prime }}$* can be defined as:

(8) $$C_{t}^{{}^{\prime }}=tanh\left(W_{c}\times h_{t-1}+W_{c}\times X_{t}+b_{c}\right)$$

The vector of the memory cell state at the current moment *C${}_{t}$* can be defined as:

(9) $$C_{t}=f_{t}\times C_{t-1}+\left(1-f_{t}\right)\times C_{t}^{{}^{\prime }}$$

The output gate layer has a peephole, and the vector it inputs is the state *C${}_{t}$* after the memory cell is updated. Therefore, the input of the output gate layer is composed of three components, which are the state *C${}_{t}$* of the current memory cell, the output of the memory cell *h${}_{t-1}$* at the last moment and the input of the memory cell *X${}_{t}$* at the current moment. *W${}_{o}$* and *b${}_{o}$* are used to represent the weight and offset of sigmoid neural network layer of the output gate, respectively. Then, the representation of the output vector of output gate layer *O${}_{t}$* can be described as:

(10) $$O_{t}=\sigma \left(W_{o}\times C_{t}+W_{o}\times h_{t-1}+W_{o}\times X_{t}+b_{o}\right)$$

The current memory cell output vector *h${}_{t}$* can be defined as:

(11) $$h_{t}=tanh\left(C_{t}\right)\times O_{t}$$

The training algorithm of LSTM including the following three steps:

Step 1: Forward calculation of the output value of each neuron in the LSTM [34].

Step 2: Reverse calculation of the error term value δ of each neuron. The back-propagation of the LSTM error term includes two directions: one is the back propagation along time, that is, the error term at each moment is calculated from the current time, and the other is to propagate the error term up to one layer [35].

Step 3: Calculate the gradient of each weight according to the corresponding error term [36].

### 3.2. LSTM-Based Error Correction Framework

The weighted least square method minimizes the sum of squares of all measurement errors and makes the positioning easy and quick. However, the estimation accuracy of the least square method is not high, and. when the observation shows a large deviation, it also causes a large deviation of the estimation result. The Kalman filter algorithm can overcome the shortcomings of the position at the adjacent time and make the filtered positioning result smoother and more accurate, thereby improving the positioning effect. To effectively reduce common errors and receiver errors, and suppress multi-path effects, the LSTM-based error correction method is proposed.

**Figure 3:**
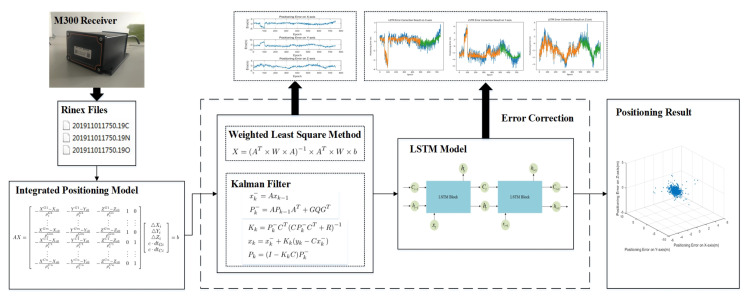
The overall flow of the LSTM-based error correction method.

The overall flow of the LSTM-based error correction method is shown in Figure 3. First, the Rinex file generated by the GNSS receiver is read. Among them, the N file means the GPS ephemeris file, the C file denotes the BDS ephemeris file and the O file represents the BDS/GPS observation file. Then, the LSTM-based error correction method is used to solve the position and finally obtain the corrected position information.

**Figure 4:**
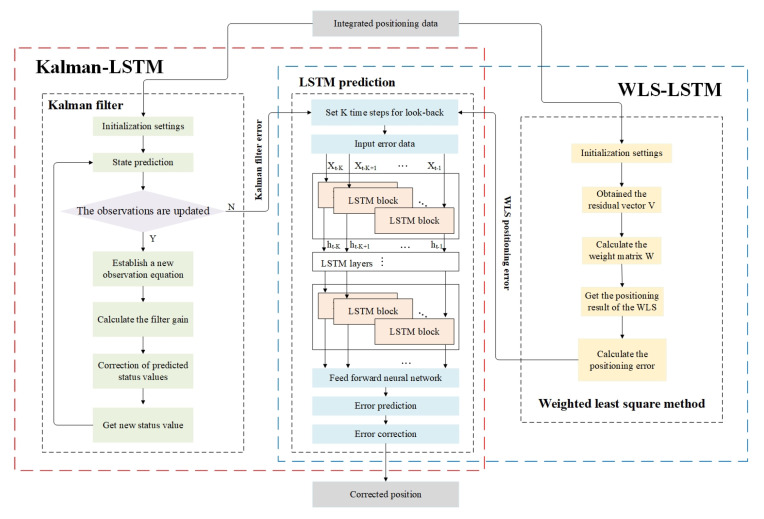
The LSTM-based error correction framework.

The LSTM-based error correction framework including WLS–LSTM and Kalman–LSTM is shown as Figure 4. The Kalman–LSTM error correction process in the red box on the left is as follows: Firstly, initial parameters are set, including sampling interval, initial state matrix, initial mean square error matrix, state transition matrix, noise driving matrix, system variance matrix, noise covariance matrix, measured value matrix and epoch number. Then, for 1 to epoch number, the vector observation matrix is constructed, followed the Kalman filter prediction and the measurement update process is performed. Then, the Kalman filter result is obtained. After that, the LSTM prediction, which uses the error result of Kalman filtering as the input of the learning network, is performed and the prediction result is obtained. Finally, the error correction is performed. The WLS–LSTM error correction process in the blue box on the right is as follows: Firstly, initial parameters are set, the same with Kalman–LSTM. Then, for 1 to epoch number, the weighted least square method is performed, including obtaining the residual vector and calculating the weight matrix. Then, the weighted least square method result is obtained. After that, the same with Kalman–LSTM, the LSTM prediction is performed, and the prediction result is obtained. Finally, the error correction is performed.

## 4. Experimental Analysis

### 4.1. Experimental Environment

#### 4.1.1. Static Experimental Environment

**Figure 5:**
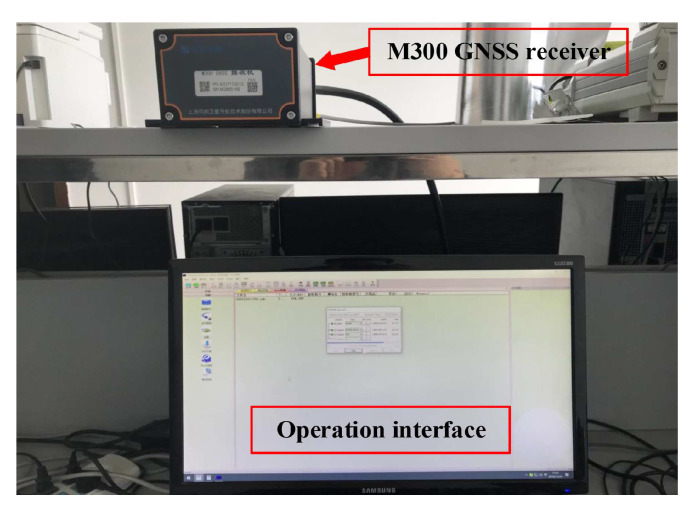
The Sinan M300 GNSS receiver experimental environment.

The integrated BDS/GPS Point Positioning was performed on a Windows PC with two 3.5 GHz Intel Xeon processors. Compass Receiver Utility software version 1.7.3 was used to operate and obtain the relevant raw data, the SPP calculation model of Integrated BDS/GPS was conducted on MATLAB and the error prediction and correction process was implemented in Python. The experimental data in this study were obtained from 11:31 to 11:59 by the Sinan M300 GNSS receiver shown in Figure 5, while the sampling interval was 60 s, the coordinates of the receiver were known and the proposed SPP calculation model and error correction methods were used to post-process the collected data.

#### 4.1.2. Dynamic Experimental Environment

**Figure 6:**
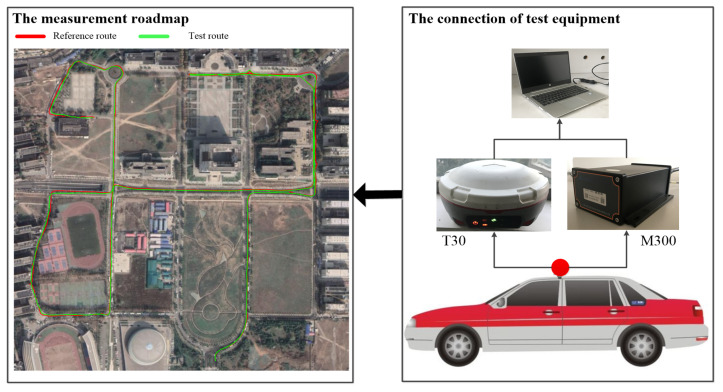
The dynamic experimental environment.

To further verify the positioning effect of the proposed method in a dynamic environment, a dynamic experiment was conducted on the campus of a university in Wuhan, China. The moving speed of the vehicle with the experimental equipment ranged from 20 to 40 km/h, and the equipment sampling interval was 1 s. In the experiment, the T30 GNSS receiver data of Sinan Navigation, whose horizontal accuracy is ±(8+1×10−6×D) mm and vertical accuracy is ±(15+1×10−6×D) mm, were used as the reference. D is the baseline length, and its unit is mm. The measurement roadmap and the connection of test equipment is shown as Figure 6.

### 4.2. Integrated BDS/GPS Point Positioning Results

#### 4.2.1. Static Positioning Results

**Figure 7:**
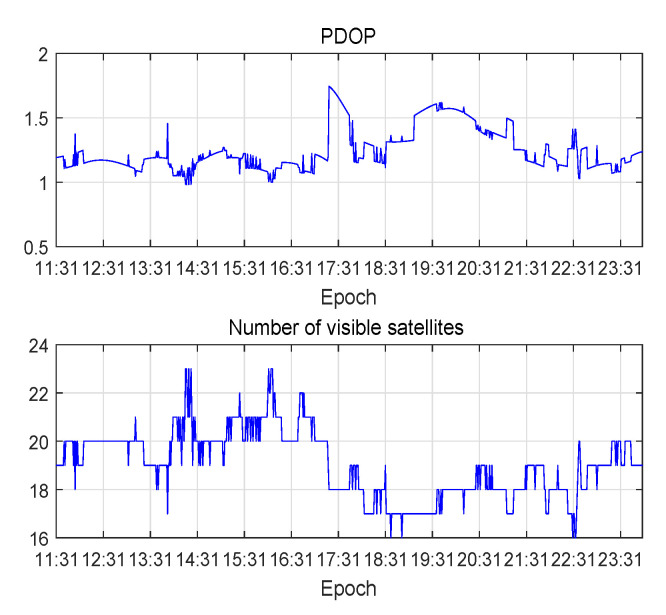
The PDOP and the number of visible satellite during the experimental period.

In satellite positioning, the position dilution of precision (PDOP), which is an important indicator for measuring the ability of satellite positioning, is usually used to represent the quality of the satellite’s geometric distribution. Generally, the more visible satellites there are, the better the satellite constellation structure, the better the satellite geometric distribution, the smaller the PDOP and the higher the positioning accuracy. The PDOP and the number of visible satellites during the experimental period are shown in Figure 7.

**Figure 8:**
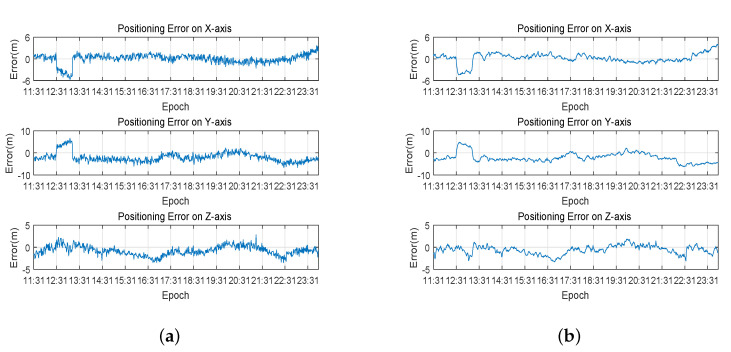
Result of standard point positioning on the three axes: (**a**) positioning error of weighted least square method; and (**b**) positioning error of Kalman filter.

The WLS and Kalman filter were performed on collected data, and the results are shown in Figure 8. The positioning errors of WLS and Kalman filter are presented in Table 1. The positioning results show that, compared to the result of WLS, Kalman filter is smoother. In terms of accuracy, the positioning root mean square error of WLS in the X-axis direction is slightly lower than the Kalman filter, while the positioning root mean square error of the Kalman filter in the Y-axis and Z-axis directions is slightly lower than the weighted least square method. One point is that the two methods have little difference in the accuracy of three-dimensional positioning, and the Kalman filter has slightly higher accuracy than WLS. In addition, the positioning error from 12:31 to 13:11 is obviously larger, but there is no significant change in PDOP and the number of visible satellites during this period. According to the analysis, this is due to the obvious large clock deviation of the BDS receiver during this period.

#### 4.2.2. Dynamic Positioning Results

**Figure 9:**
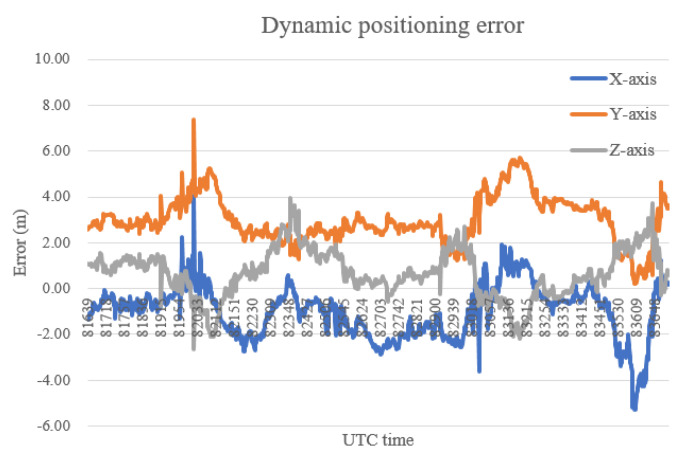
The dynamic positioning error.

The dynamic positioning results are shown in Figure 9, and the dynamic positioning errors are presented in Table 2, from which it can be found that the 3D error of dynamic positioning is slightly higher than that of static positioning.

### 4.3. Error Prediction Results

#### 4.3.1. Static Prediction Results

To reduce the common errors, receiver errors and multi-path effects more effectively on the basis of WLS and Kalman filter, the LSTM-based error prediction was performed on the integrated positioning. The prediction result can be used to correct the errors caused by multi sources.

**Figure 10:**
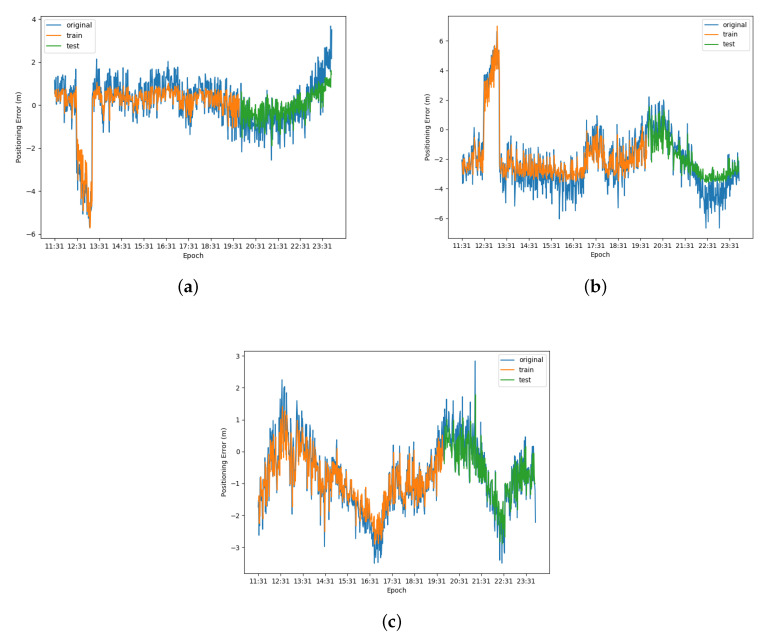
Result of LSTM error prediction performed on the weighted least square method: (**a**) result on X-axis; (**b**) result on Y-axis; and (**c**) result on Z-axis.

**Figure 11:**
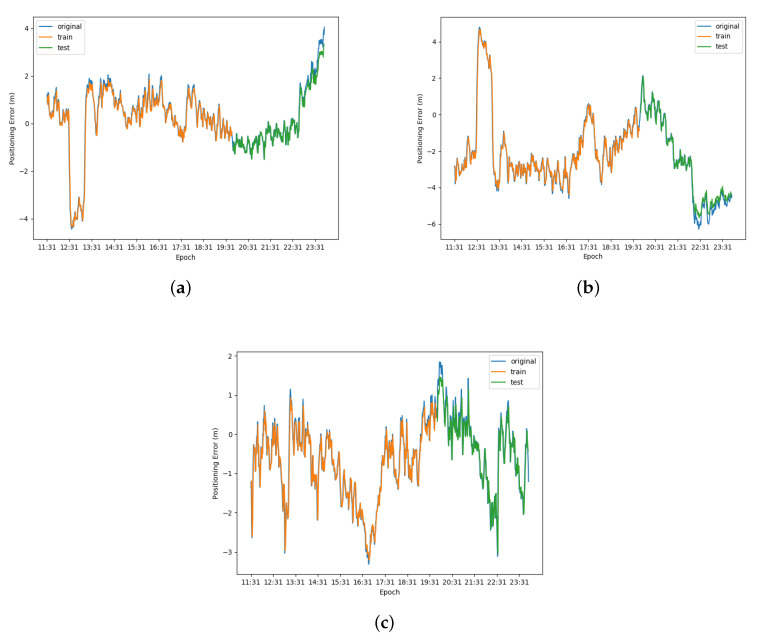
Result of LSTM error prediction performed on Kalman filter: (**a**) result on X-axis; (**b**) result on Y-axis; and (**c**) result on Z-axis.

The prediction results of LSTM-based error prediction performed on WLS is presented in Figure 10, while that performed on Kalman filter is shown in Figure 11. The error data were divided into training set and testing set, and the ratio of the training set to the testing set was 2:1. In Figure 10 and Figure 11, the blue curves denote the original data, the orange curves represent the prediction of training set and the green curves mean the prediction testing set. The root mean square errors (RMSE) and correlation coefficient of the prediction of two methods on three axes are shown in Table 3. From the prediction results, the root mean square error of WLS is higher and the correlation coefficient is smaller than the Kalman filter in both training set and testing set on the three axes, which illustrates that the performance on the prediction of Kalman filter is better than the prediction of WLS on three axes.

#### 4.3.2. Dynamic Prediction Results

**Figure 12:**
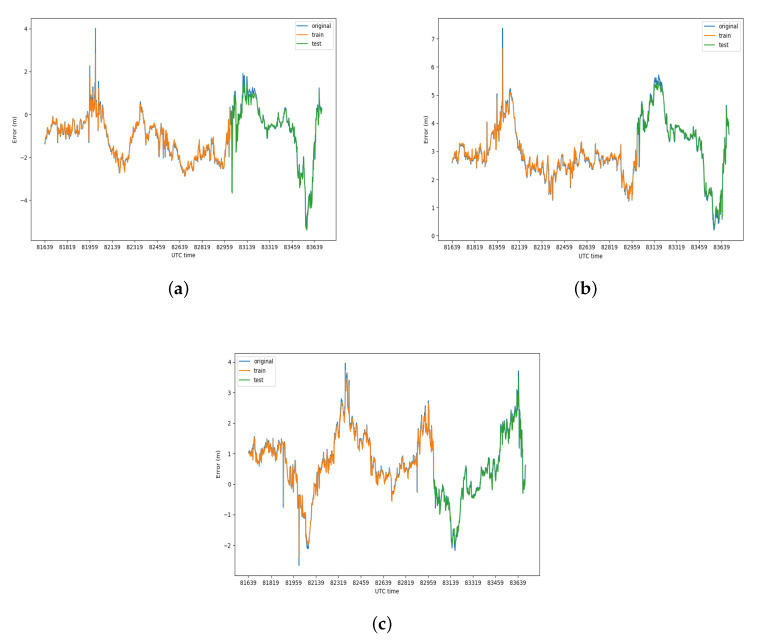
Result of dynamic LSTM error prediction: (**a**) result on X-axis; (**b**) result on Y-axis; and (**c**) result on Z-axis.

The error prediction based on LSTM was also performed on dynamic experimental data. The prediction result is presented in Figure 12 and prediction errors are shown in Table 4. As in the static prediction, the error data were divided into training set and testing set, and the ratio of the training set to the testing set was 2:1. Compared with the prediction errors of the static prediction, the effect of dynamic prediction on the Y-axis and Z-axis is better than the two static methods, while, on the X-axis, its effect is better than the static prediction of WLS method but slightly worse than the static prediction of Kalman filter on the testing set.

### 4.4. Error Corrected Positioning Results and Evaluations

#### 4.4.1. Static Corrected Results

**Figure 13:**
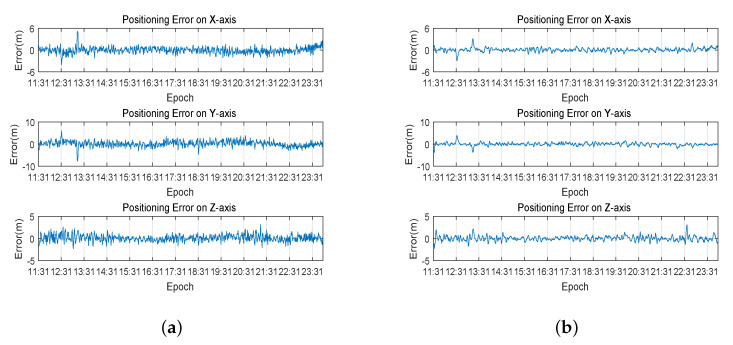
Result of LSTM-based error correction method on the three axes: (**a**) positioning error of WLS–LSTM; and (**b**) positioning error of Kalman–LSTM.

The prediction results were used to achieve the error correction. The LSTM error correction performed on the weighted least square method is called WLS–LSTM error correction, while the LSTM error correction performed on Kalman filter is called Kalman–LSTM error correction. The result of WLS–LSTM error correction and Kalman–LSTM error correction are presented in Figure 13. The positioning errors of the two error correction methods are shown in Table 5. The root mean square error of the Kalman–LSTM error correction method in the X-axis, Y-axis and Z-axis are 0.523, 0.705 and 0.554 m, respectively. The root mean square error of the WLS–LSTM error correction method in the X-axis, Y-axis and Z-axis are 0.880, 1.345 and 0.770 m, respectively. The experimental result illustrates that the positioning result of Kalman–LSTM error correction method is smoother and more accurate than the WLS–LSTM error correction method.

**Figure 14:**
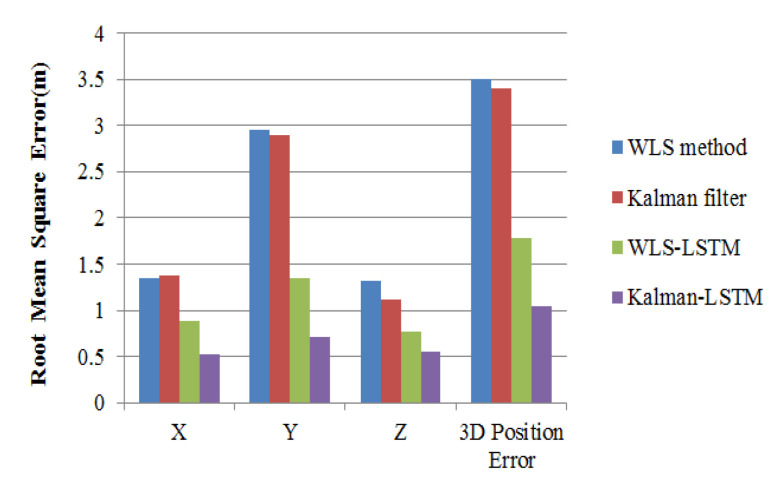
The position error of four different methods.

The positioning results of the four different methods are presented in Figure 14. From the positioning results, compared to the positioning methods without LSTM correction, the positioning error of LSTM-based error correction methods is significantly reduced on three axes. The Kalman–LSTM error correction is the most accurate method for its positioning error is significantly smaller than the other methods. It is worth mentioning that the point positioning error of Kalman–LSTM error correction method can almost reach the sub-meter level, which is 1.038 m from the experimental result, while the point positioning error of WLS–LSTM error correction method, Kalman filter and WLS method are 1.782, 3.406 and 3.498 m, respectively. Moreover, the positioning error from 11:31 to 11:59, which is obviously larger, is effectively reduced by the proposed error correction method.

#### 4.4.2. Dynamic corrected results

**Figure 15:**
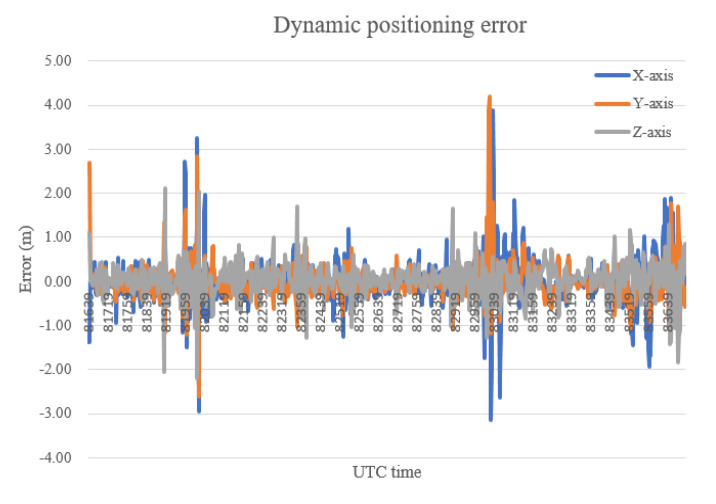
The corrected dynamic positioning results.

The corrected dynamic positioning results of proposed method are shown in Figure 15, while the corrected dynamic positioning errors are presented in Table 6. It can be seen from the experimental error results that it is obvious that the positioning error corrected by the proposed method in a dynamic scene is smaller on the X-axis, Y-axis and Z-axis than the two methods in a static scene, which illustrates that the error correction method may achieve better results in a dynamic scene.

#### 4.4.3. Evaluations

**Figure 16:**
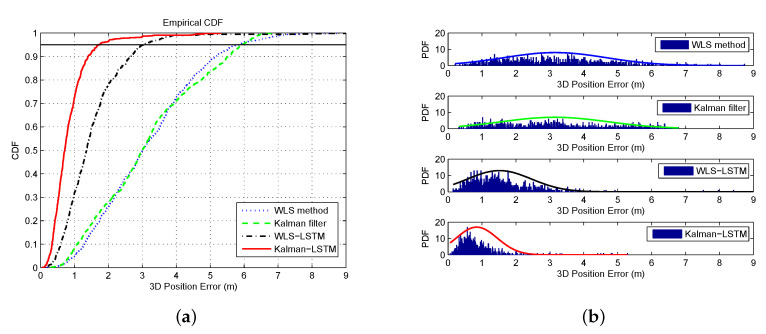
The CDF and PDF of four different methods: (**a**) CDF; and (**b**) PDF.

To further evaluate the performance of each method, the cumulative distribution function (CDF) and probability density function (PDF) of positioning errors were used as evaluation indicators. The CDF is used to describe the probability that the random variable X falls within a certain range and PDF is a function that describes the probability of the output value of the random error, near a certain point of determination [37,38]. The CDF and PDF of four different methods in a static scene are presented in Figure 16. It can be seen from the CDF that the error at 95% confidence of Kalman–LSTM is obviously better than the other methods, the WLS–LSTM is also better than Kalman filter and WLS, while the Kalman filter and WLS are almost the same. As shown in Figure 16, the PDF of the position error in Kalman–LSTM is the most concentrated, especially most position errors appear within 1 m and only a few position errors are above 2 m. The PDF of the position error in WLS–LSTM is also more concentrated than Kalman filter and WLS: most position errors concentrate within 2 m and a few are distributed 4 m away. The position errors of Kalman filter and WLS are more dispersed, and Kalman filter is a little more concentrated than WLS. In addition, the position errors of Kalman filter appear within 7 m while WLS appear within 9 m. One can draw the conclusion from the results of CDF and PDF that the LSTM-based has a better performance than Kalman filter and WLS method on integrated BDS/GPS positioning for the higher accuracy and more concentrated position errors.

**Figure 17:**
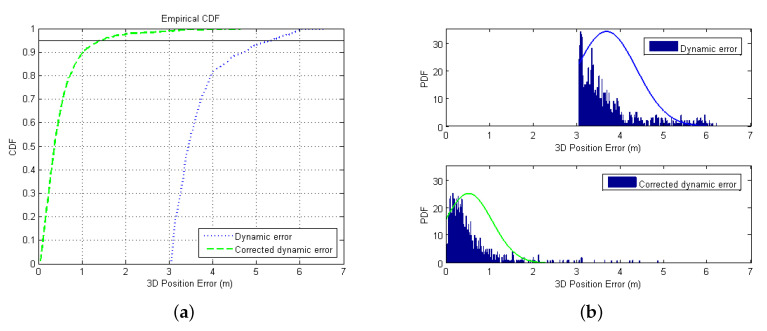
The CDF and PDF of dynamic error: (**a**) original error; and (**b**) corrected error.

The CDF and PDF results in a dynamic scene are presented in Figure 17. It can be seen from the CDF that the corrected error (within 1.5 m) at 95% confidence is significantly improved compared to before correction (above 5 m) and better than the effect in the static scene. As shown in Figure 17b, the dynamic errors before correction are all above 3 m, concentrated between 3 and 4 m, and the maximum error is less than 7 m. Most of the corrected errors are within 1 m, and the maximum error is less than 5 m, which is better than the WLS–LSTM method in the static error. Compared with the Kalman–LSTM method, the overall effect is better, but the proportion of errors above 2 m is higher. It can be concluded that the proposed error improvement method also has a good effect in dynamic scenes and may even be better than the effect in static scenes.

## 5. Conclusion

This paper provides a novel approach to the standard point positioning of integrated BDS/GPS. First, the different error sources of standard point positioning of integrated BDS/GPS are elaborated. Among them, some error sources are difficult to estimate during the positioning process. In some current positioning methods, they are also difficult to obtain very effectively reduced or suppressed. Therefore, an LSTM-based error prediction and correction method is proposed in this paper. On the basis of the traditional positioning method, the data with multiple error sources are learned and predicted, and the prediction results are used for error correction to effectively reduce and suppress the error containing multiple error sources.

The four methods, namely weighted least square method, Kalman filter, WLS–LSTM and Kalman–LSTM, were tested and compared based on the measured data. It turns out that the LSTM recurrent neural network-based error correction method has greatly improved the accuracy of standard point positioning. The proposed Kalman–LSTM error correction method achieves the best performance, whose point positioning error can almost reach the sub-meter level under the experimental environment. Furthermore, through the error correction method, the positioning error caused by various factors such as the receiver clock deviation can be effectively suppressed. The greater the error is, the more the Kalman–LSTM can contribute to the positioning improvement of the methods without LSTM error correction. In addition, the experimental results in dynamic scenes also show that the proposed LSTM-based error correction method can effectively improve the positioning accuracy.

As for future work, parameter tuning of the LSTM network structure can be developed, and the models in different environments and scenarios can be trained to adapt the method to the navigation and positioning requirements in different environments and scenarios.

## Figures and Tables

**Table 1:** Positioning errors of the two methods.

Positioning	Root Mean Square Error (m)			3D Position
Methods	X	Y	Z	Error (m)
WLS method	1.347	2.945	1.322	3.498
Kalman filter	1.379	2.897	1.123	3.406

**Table 2:** Dynamic positioning errors.

Axis	X	Y	Z	3D
Positioning Error (m)	1.5460	3.1694	1.2459	3.7399

**Table 3:** Prediction errors and correlation coefficient of the two methods.

Predicted Methods	RMSE of Training (m)			RMSE of Testing (m)			Correlation Coefficient		
	X	Y	Z	X	Y	Z	X	Y	Z
WLS method	0.77	1.17	0.65	0.90	1.26	0.74	0.7580	0.7842	0.6985
Kalman filter	0.27	0.34	0.26	0.28	0.34	0.32	0.9262	0.9439	0.8303

**Table 4:** Prediction errors and correlation coefficient of dynamic experimental data.

	RMSE of Training (m)			RMSE of Testing (m)			Correlation Coefficient		
Axis	X	Y	Z	X	Y	Z	X	Y	Z
Positioning Error	0.27	0.22	0.23	0.34	0.21	0.22	0.9072	0.9097	0.9396

**Table 5:** Positioning errors of error correction methods.

Error Correction Methods	Root Mean Square Error (m)			3D Position Error (m)
	X	Y	Z	
WLS–LSTM	0.880	1.345	0.770	1.782
Kalman–LSTM	0.523	0.705	0.554	1.038

**Table 6:** Error corrected dynamic positioning errors.

Axis	X	Y	Z	3D
Positioning Error (m)	0.4949	0.4289	0.3641	0.7493

## Abbreviations

The following abbreviations are used in this manuscript:

BDS	BeiDou Navigation Satellite System
GPS	Global Positioning System
SPP	Standard Point Positioning
LSTM	Long Short-Term Memory
WLS	Weighted Least Square
RTK	Real-time Kinematic
PPP	Precision Point Positioning
WP-TD	Two-dimensional Moving Weighted Average Processing
DVL	Doppler Velocity Log
RAFSTKF	Robust Adaptive Federated Strong Tracking Kalman Filter
RNN	Recurrent Neural Network
BDT	Bei Dou Navigation Satellite System Time
UTC	Coordinated Universal Time
GPST	GPS Time
CGCS2000	China Geodetic Coordinate System 2000
WGS-84	World Geodetic Coordinate System 1984
ITRF	International Terrestrial Reference Frame
IERS	International Earth Rotation Service
PDOP	Position Dilution of Precision
RMSE	Root Mean Square Errors
CDF	Cumulative Distribution Function
PDF	Probability Density Function
